# Nanoparticle-Mediated Local Delivery of an Antisense *TGF-β1* Construct Inhibits Intimal Hyperplasia in Autogenous Vein Grafts in Rats

**DOI:** 10.1371/journal.pone.0041857

**Published:** 2012-07-30

**Authors:** Da-Xin Sun, Zhen Liu, Xiao-Dong Tan, Dong-Xu Cui, Bao-Sheng Wang, Xian-Wei Dai

**Affiliations:** Department of General Surgery, Shengjing Hospital of China Medical University, Shenyang, Liaoning Province, China; National Center for Scientific Research Demokritos, Greece

## Abstract

**Background:**

Intimal hyperplasia is one of the most important causes of vascular graft failure. Numerous studies have correlated transforming growth factor-β1 (TGF-β1) with extracellular matrix (ECM) deposition, a hallmark of intimal thickening.

**Principal Findings:**

In the present study, we performed immunohistochemistry, RT-PCR, and Western blot to examine the dynamic expression of TGF-β1, TGF-β1 receptor type I (TGF-β RI), matrix metalloproteinase-1 (MMP-1) and tissue inhibitor of metalloproteinase-1 (TIMP-1) during intimal hyperplasia in grafted veins of a rat model generated by grafting a portion of the right internal jugular vein to the ipisiliary caroid artery. Additionally, we determined whether nanoparticle-mediated delivery of a *TGF-β1* antisense-expressing construct prevented TGF-β1 expression and intimal hyperplasia in grafted veins. In grafted veins, the expression of TGF-β1 significantly increased on day 3 after transplantation, peaked on day 7, slightly decreased on day 14, and returned to baseline levels on day 28. The positive expression of TGF-β RI in grafted veins remarkably increased on day 7, peaked on day 14, and decreased thereafter. MMP-1 expression decreased significantly, while TIMP-1 expression increased, significantly on days 14 and 28. Nanoparticle-mediated delivery of a *TGF-β1* antisense-expressing construct down-regulated TGF-β1 expression and inhibited intimal hyperplasia in grafted veins.

**Conclusions:**

Our findings provide further evidence that TGF-β1 plays an integral role in the development of intimal hyperplasia after vascular injury. Nanoparticle-mediated delivery of a *TGF-β1* antisense-expressing construct is a feasible strategy to target TGF-β1-induced intimal thickening.

## Introduction

Lifestyle changes and aging have greatly increased the global prevalence of coronary artery disease and peripheral arterial occlusive disease over the past several decades. Artery bypass surgery is the standard revascularization strategy, while grafting of autogenous veins, especially the great saphenous vein, is the most widely used technique in both coronary artery and peripheral artery bypass procedures. Despite the excellent results achieved with autogenous vein grafts for artery bypass, a considerable percentage of these patients develop stenosis or occlusion within one year [1]. Elucidating the mechanisms underlying vein graft stenosis may help overcome autogenous vein graft failure.

It has been shown that intimal hyperplasia, which is characterized by the migration and proliferation of vascular smooth muscle cells (VSMCs) with concomitant deposition of extracellular matrix (ECM), is one of the most important causes of vascular graft failure within the first year after operation [2]. The bulk of neointima (∼90%) is composed of ECM, while VSMCs account for only 11% of neointimal restenosis lesions [1], [3]. A large body of evidence shows that matrix metalloproteinases (MMPs) and tissue inhibitors of metalloproteinases (TIMPs) participate in intimal hyperplasia by regulating VSMC proliferation and migration [4], [5]. However, the mechanisms by which MMPs and TIMPs are regulated during neointima formation after vascular injury is not completely understood.

Transforming growth factor-β1 (TGF-β1) has been identified as a critical regulator of intimal hyperplasia after vascular injury [6]. Up-regulation of TGF-β1 mRNA and protein expression has been observed during progressive neointimal thickening [7], [8]. Administration of recombinant TGF-β1 or overexpression of this growth factor enhances intimal hyperplasia [9], [10], whereas inhibition of TGF-β1 signaling suppresses intimal hyperplasia in animal models [3], [11]–[13]. In addition, targeted disruption of TGF-β-Smad3 signaling diminishes matrix deposition in response to vascular injury [14], suggesting that TGF-β1 facilitates ECM deposition during intimal hyperplasia.

Given the central role of TGF-β1 in the regulation of ECM deposition during intimal hyperplasia after vascular injury, multiple strategies have been developed to antagonize TGF-β1 signaling to inhibit intimal hyperplasia [6]. However, several clinical trials evaluating the efficacy of oral TGF-β1 inhibitors for prevention of restenosis obtained negative results [6], [15]. Results from these studies indicated that systemic administration of TGF-β1 inhibitors was not able to attain sufficient local concentrations at injury sites, emphasizing the need for local delivery to achieve and sustain optimal vascular levels of TGF-β1 inhibitors. Traditional local delivery systems have been shown to lack sustained delivery and adversely affect cell viability. Therefore, sustained-release, biodegradable nanoparticles represent a potential alternative for prolonged local delivery of agents [16].

In the present study we determined the dynamic expression of TGF-β1, TGF-β1 receptor type I (TGF-β RI), MMP-1, and TIMP-1 in grafted veins in a rat model. We also examined the impact of nanoparticle-mediated delivery of a *TGF-β1* antisense-expressing construct on TGF-β1 expression and intimal hyperplasia in grafted veins. This study will provide further insight into the role of TGF-β1 in the development of intimal hyperplasia after vascular hyperplasia.

## Materials and Methods

### Preparation, Characterization, and Delivery of Nanoparticles Carrying a TGF-β1 Antisense-expressing Construct

Professor Lu Yinglin at the Institute of Basic Medical Sciences of Academy of Military Medical Sciences provided the eukaryotic expression vector pMAMneo-Anti-TGF-β1, which expresses TGF-β1 antisense transcripts. Silica nanoparticles were prepared as previously described [17]. Briefly, cyclohexane, deionized water, and ethyl silicon were mixed. After aqueous ammonia was added, the mixture was allowed to react for 24 h at room temperature. The reactant was then centrifuged at 10,000 rpm for 10 min, rinsed with acetone, and dried in a vacuum. The obtained nanoparticles (0.09 g) were dissolved in 6 mL of deionized water, dispersed by sonication for 2 h, and autoclaved. Prior to use, 50 mL of suspended nanoparticles were added to 4 mg of pMAMneo-Anti-*TGF-β1* and 1 mol/L of NaCl solution, and mixed for 15 min at room temperature. The size and morphology of the nanoparticles were observed by scanning electron microscopy. The concentration of DNA loaded onto the nanoparticles was determined as follows: 1) a portion of the nanoparticle suspension was centrifuged at 10,000 rpm for 5 min; 2) the nanoparticles were incubated with TE buffer (pH  = 10.0) for 3 min to elute DNA; and 3) the DNA content in the eluate was determined by ultraviolet spectrophotometry.

**Figure 1:**
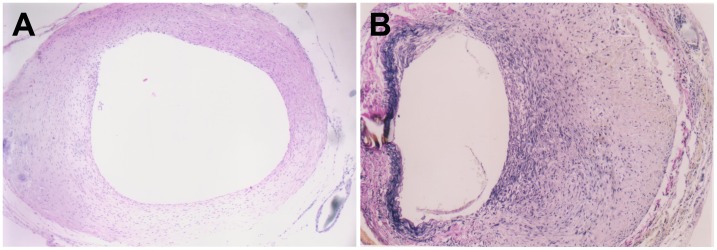
Neointimal thickening in grafted veins. Photomicrographs showing representative cross sections of an H&E-stained grafted vein on day 7 (A) and Verhoffe-Van Gieson-stained grafted vein on day 14 (B). (*n*  = 6, magnification ×200).

**Table 1:** Changes in intimal and wall thickness of grafted veins.

Group	Intimal thickness (µm)	*P*	Wall thickness (µm)	*P*
Control	6.0±1.3		138±11	
Day 3	7.2±2.1	0.2615	142±18	0.6523
Day 7	18.5±2.7*	<0.001	405±31*	<0.001
Day 14	45.2±4.8*	<0.001	389±36*	<0.001
Day 28	40.9±3.5*	<0.001	400±32*	<0.001

Data shown are expressed as mean ± SD (*n*  = 6).* vs. Control.

### Creation of a Rat Model of Autogenous Vein Grafting

Twenty-four male Wistar rats weighing 250–300 g (provided by the Laboratory Animal Center of Shengjing Hospital of China Medical University) were anesthetized by intraperitoneal injection of chloral hydrate (30 mg/kg). A longitudinal incision was made along the median line of the anterior neck under an operating microscope. A portion of the right internal jugular vein (4–5 mm in length) was anastomosed to the ipsilateral carotid artery and the incision was closed. The post-operative rats were randomly divided into four groups for testing at different time points (3, 7, 14, and 28 d; n  = 6 for each time point). An additional 45 male Wistar rats were used for nanoparticle-mediated gene delivery experiments. The operating procedure was performed essentially as described above, but a catheter was inserted into the proximal end of the vein for local delivery of pMAMneo-Anti-*TGF-β1*-loaded nanoparticles before the proximal end of the vein was anastomosed (*n*  = 15). Equal volumes of empty vector (pMAMneo)-loaded nanoparticles or normal saline were delivered as controls (*n*  = 15 for each control condition). The fluid was kept in the lumen for 30 min, aspirated, and applied onto the external surface of the grafted vein. After local delivery, the proximal end of the vein was anastomosed to the artery and the incision was closed. The animals were sacrificed after 2 weeks. The experiments were approved by the ethics committee of Shengjing Hospital of China Medical University and conducted in accordance with their guidelines.

### Histology

Right internal jugular vein segments were cut and fixed in 4% paraformaldehyde for 24 h, dehydrated, embedded in paraffin, and cut into serial sections. Contralateral normal vein from the same rat served as the control. Sections were stained with hematoxylin and eosin (H&E) for general tissue structure and Verhoffe-Van Gieson stain for elastic and collagen fibers. Neointima was defined as the region between the lumen and the internal elastic lamina. Five segments of each vein were selected to determine the thickness of intima and vein wall by a technician using the MetaMorph BX41 image analysis system (Olympus, Japan). All the measurements were made in a blind manner.

**Figure 2:**
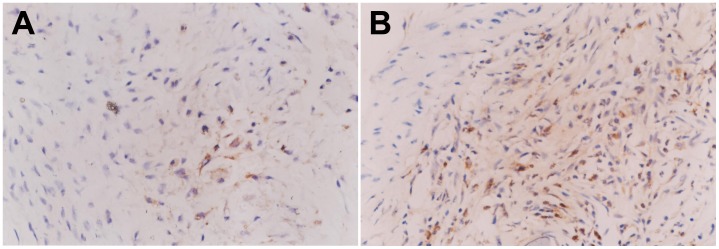
Immunohistochemical analysis of TGF-β1 and TGF-β RI expression in grafted veins. Photomicrographs showing representative sections of immunostained grafted veins of TGF-β1 on day 7 (A) and TGF-β RI on day 14 (B). (*n*  = 6, magnification ×400).

**Table 2:** Immunohistochemical staining for TGF-β1 and TGF-β R1 in grafted veins (n = 6).

Group	TGF-β1				P*	TGF-β R1				P*
	−	+	++	+++		−	+	++	+++	
Control	5	1	0	0		6	0	0	0	
Day 3	1	3	1	1	0.026	5	1	0	0	1.000
Day 7	0	0	4	2	0.002	0	4	2	0	0.002
Day 14	0	1	3	2	0.009	0	5	1	0	0.002
Day 28	4	2	0	0	1.000	0	4	1	1	0.002

0, negative staining (−); 1–4, weak staining (+); 5–8, moderate staining (++); and 9–12, intense staining (+++). The Fisher exact test was used for intergroup comparisons of immunohistochemistry scores. P-value was calculated vs. control.

### Immunohistochemistry

Deparaffinized sections were treated with 3% H_2_O_2_ for 10 min to inactivate peroxidases, heated in 10 mM citrate buffer at 121°C for 30 min for antigen retrieval, blocked in 5% normal serum for 20 min, and incubated with a primary polyclonal anti-TGF-β1 (dilution, 1∶150 in phosphate buffered saline (PBS); sc-146, Santa Cruz Biotechnology, Santa Cruz, CA, USA; antibody recognizes both precursor and mature forms of TGF-β1), anti-TGF-βRI (dilution, 1∶100 in PBS; sc-398, Santa Cruz Biotechnology), anti-MMP1 (dilution, 1∶100 in PBS; sc-30069, Santa Cruz Biotechnology), or anti-TIMP-1 (dilution, 1∶50 in PBS; sc-5538 Santa Cruz Biotechnology) antibody overnight at 4°C. After three extensive washes with PBS, sections were incubated with a biotin-conjugated secondary antibody (dilution, 1∶2000 in PBS; sc-2040, Santa Cruz Biotechnology) for 20 min at 32°C. After further incubation with horseradish peroxidase (HRP)-labeled streptavidin, antibody binding was visualized with diaminobenzidine and sections were counterstained with hematoxylin. For negative controls, primary antibody was replaced with PBS alone. The presence of brown granules in the membrane, cytoplasm or nucleus was considered positive staining. Five visual fields were randomly selected from each section to count 200 cells to evaluate the percentage of positively stained cells and staining intensity. The percentage of positively stained cells was scored from 0 to 4: <1%, 1–10%, 11–50%, 51–80%, and >80%, respectively. Staining intensity was scored from 0 to 3 according to the extent of cell coloration. The product of the score of the percentage of positively stained cells and that of staining intensity was then calculated to evaluate staining positivity: 0, negative staining (−); 1–4, weak staining (+); 5–8, moderate staining (++); and 9–12, intense staining (+++).

**Figure 3:**
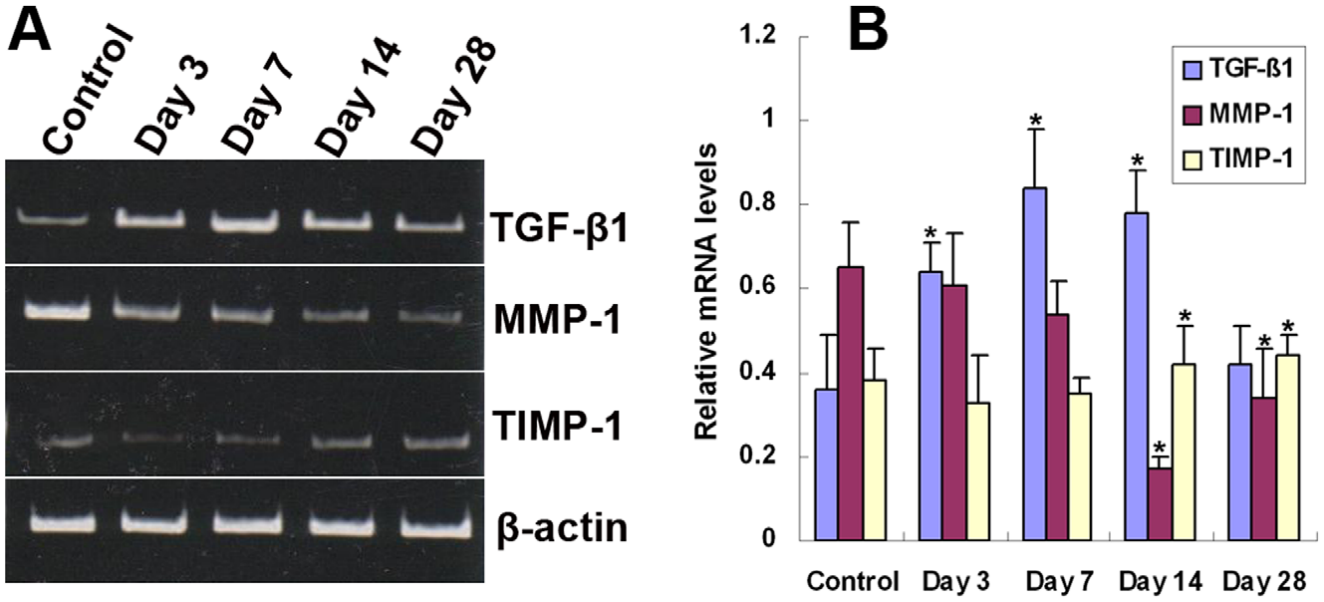
Temporal expression of *TGF-β1*, *MMP-1,* and *TIMP-1* mRNA in grafted veins. Total RNA was extracted from grafted veins and reverse transcribed into cDNA. The relative levels of *TGF-β1*, *MMP-1*, and *TIMP-1* transcripts in grafted veins on days 3, 7, 14, and 28 were determined by PCR. (A) Representative RT-PCR products. (B) Quantitative analysis of RT-PCR products (*n*  = 6). *P<0.05 vs. Control.

**Figure 4:**
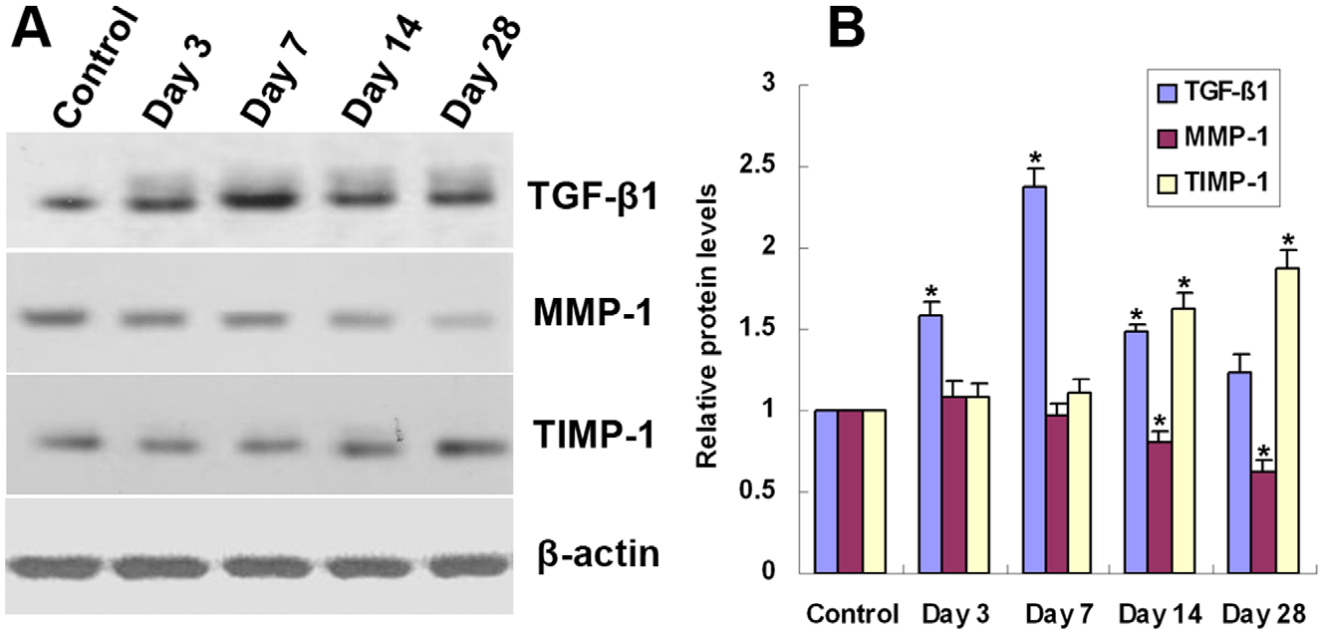
Temporal expression of TGF-β1, MMP-1, and TIMP-1 protein in grafted veins. Western blot was performed to determine the expression of TGF-β1, MMP-1 and TIMP-1 in grafted veins on days 3, 7, 14, and 28. β-actin was used as a loading control. (A) Representative Western blot image. (B) Quantitative analysis of Western blot images (*n*  = 6). *P<0.05 vs. Control.

### Western Blotting

Tissue samples from vein grafts were homogenized in RIPA lysis buffer, and proteins were resolved by 15% SDS-polyacrylamide gel electrophoresis and transferred onto a nitrocellulose membrane (Hybond-ECL, Amersham Pharmacia Biotech, Uppsala, Sweden). The membrane was blocked in TBS (20 mM Tris-HCl, 150 mM NaCl, pH  = 7.4) containing 5% skim milk, incubated with anti-TGF-β1 (sc-146), anti-MMP-1 (sc-30069) or anti-TIMP-1 (sc-5538) primary antibody (dilution 1∶400, Santa Cruz Biotechnology) overnight at 4°C, and then incubated with a HRP-conjugated secondary antibody (dilution, 1∶2000; sc-2004, Santa Cruz Biotechnology) for 1 h at room temperature. Protein bands were visualized using enhanced chemiluminescence. β-actin was used as an equal loading control. Signals of the protein bands were scanned and quantified using the Meta Morph/BX41 image analysis system (Olympus, Tokyo, Japan), and the ratios of the intensity of protein bands versus controls were calculated.

**Figure 5:**
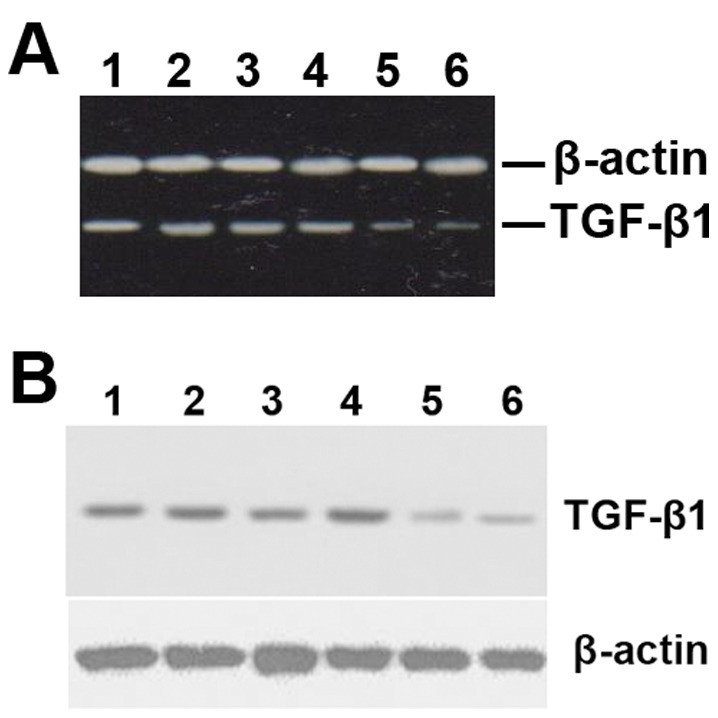
Nanoparticle-mediated delivery of a *TGF-β1* antisense-expressing construct down-regulates the expression of TGF-β1 mRNA and protein in grafted veins. Following local delivery of empty vector-loaded nanoparticles (lanes 1 and 2), normal saline (lanes 3 and 4), or pMAMneo-Anti-*TGF-β1*-loaded nanoparticles (lanes 5 and 6), the grafted veins were collected and RT-PCR and Western blot analyses performed (*n*  = 15). β-actin was used as a loading control. The RT-PCR (A) and Western blot (B) images are representive of at least three different independent experiments, all of which gave similar results.

**Table 3:** Immunohistochemical staining for MMP1 and TIMP1 in grafted veins after nanoparticle-mediated delivery of the TGF-β1 antisense-expressing construct (n = 15).

Group	MMP1				P*	TIMP1				P*
	-	+	++	+++		-	+	++	+++	
Normal saline	2	10	3	0		0	5	8	2	
Empty vector	1	9	4	0	1.000	1	4	10	0	0.526
Anti-TGF-β1	0	4	8	3	0.015	1	11	3	0	0.034

0, negative staining (−); 1–4, weak staining (+); 5–8, moderate staining (++); and 9–12, intense staining (+++). The Fisher exact test was used for intergroup comparisons of immunohistochemistry scores. *P-value calculated vs. normal saline.

**Figure 6:**
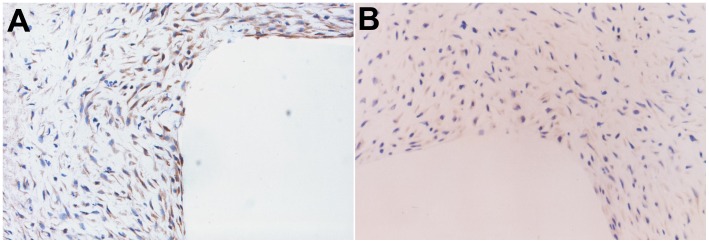
Immunohistochemical analysis of MMP1 and TIMP1 expression in grafted veins after nanoparticle-mediated delivery of the *TGF-β1* antisense-expressing construct. Photomicrographs showing representative sections of grafted veins immunostained for MMP1 (A) and TIMP1 (B) expression (*n*  = 15, magnification ×400).

**Figure 7:**
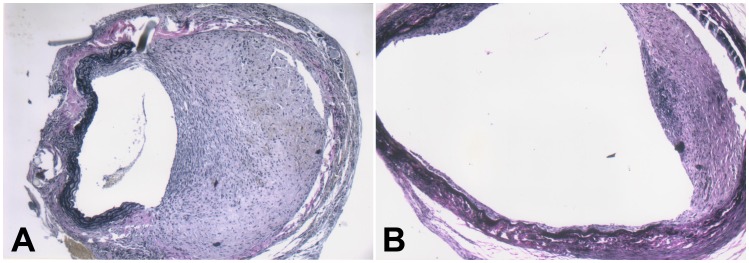
Nanoparticle-mediated delivery of a TGF-β1 antisense-expressing construct inhibits intimal hyperplasia in grafted veins. Following local delivery of empty vector-loaded nanoparticles, normal saline or pMAMneo-Anti-*TGF-β1*-loaded nanoparticles, the grafted veins were collected on day 14 after grafting and Verhoffe-Van Gieson staining performed. Representative images of Verhoffe-Van Gieson-stained grafted veins treated with empty vector-loaded nanoparticles, with the intimal thickness of 47.3±6.4 µm (A) or pMAMneo-Anti-*TGF-β1*-loaded nanoparticles with the intimal thickness of 20.5±4.7 µm (B) are shown. (*n* = 15, magnification ×100).

### RT-PCR

Total RNA was isolated from vein graft samples using TRIzol reagent (Gibco, Grand Island, NY), and cDNA was synthesized and used for PCR amplification. PCR to detect the expression of *TGF-β1*, *MMP-1*, and *TIMP-1* in vein grafts was performed as follows: 95°C for 5 min; 35 cycles of 94°C for 30 s, 56–60°C for 30 s and 72°C for 60 s; and 72°C for 10 min. PCR products were resolved on agarose gels and visualized after ethidium bromide staining under UV illumination. Amplification of *β-actin* was used as a loading control. The sequences of the primers used were as follows: *TGF-β1* (324 bp) forward: 5′-GCC CTG GAC ACC AAC TAT TGC-3′ and reverse: 5′-GGA GCG CAC GAT CAT GTT GG-3′; *MMP-1* (251 bp) forward: 5′-GGT GGC CAG AAT AGC TGA ATG-3′ and reverse: 5′-GCG TTT TGA TAT GCC C-3′; *TIMP-1* (900 bp) forward: 5′-ACC CAC AGA CGG CCT TCT GCA ATT C-3′ and reverse: 5′-GGC TAT CTG GGA CCG CAG GGA CTG C-3′; and *β-actin* (540 bp) forward: 5′-GTG GGC CGC TCA AGG CAC CAA-3′ and reverse: 5′-CTT TAG CAC GCA CTG TAG TTT CTC-3′. Bands were quantified by densitometric scanning using the Meta Morph/BX41 image analysis system (Olympus, Tokyo, Japan).

### Statistical Analysis

Numerical data are expressed as the mean ± standard deviation (SD) and statistical significance analysis performed using student’s *t*-test. The Fisher exact test was used for intergroup comparisons of immunohistochemistry scores. *P-*values <0.05 were considered statistically significant.

## Results

### Intimal Hyperplasia in Grafted Veins

In normal veins, the intima was intact and thin throughout the entire experimental course. In vein grafts, no remarkable intimal thickening was observed on day 3 after grafting. However, on day 7 post-surgery, the intima thickened significantly and consisted largely of abundant ECM with embedded VSMCs (Fig. 1A). Intimal thickening became most obvious on day 14, with ruptured elastic fibers visible in the region of intimal hyperplasia (Fig. 1B). Compared to normal veins, both intimal and wall thickness of grafted veins were significantly increased on days 7, 14 and 28 (Table 1), although no significant differences were observed on day 3.

### Dynamic Expression of TGF-β1 and TGF-β R1 in Grafted Veins

The expression of TGF-β1 and TGF-β R1 in grafted veins was assessed by immunohistochemistry. As shown in Figure 2, positive staining for TGF-β1 was mainly found in the cytoplasm of VSMCs in the media and neointima, and scarcely found in the ECM or adventitial connective tissue. In grafted veins, the expression of TGF-β1 protein significantly increased on day 3 after grafting, peaked on day 7, slightly decreased on day 14, and returned to baseline levels on day 28 (Table 2). The expression of TGF-β1 protein in grafted veins was significantly higher on days 3, 7, and 14 (*Ps* <0.05 for all) compared to control veins, but there was no significant difference in TGF-β1 levels on day 28. Positive staining for TGF-β RI expression was detected in the membrane, cytoplasm, and nucleus of VSMCs. The expression of TGF-β RI in grafted veins increased on day 7, peaked on day 14, and decreased thereafter (Table 2).

### Temporal Expression of TGF-β1, MMP-1, and TIMP-1 mRNAs and Proteins in Grafted Veins

The temporal expression of TGF-β1, MMP-1, and TIMP-1 mRNA and protein in grafted veins was evaluated by RT-PCR and Western blotting, respectively (Figs. 3 and 4). Similar to the immunohistochemistry results, the expression of TGF-β1 mRNA and protein in grafted veins significantly increased on day 3 after implantation compared to the control, peaked on day 7, slightly decreased on day 14, and markedly decreased on day 28. In contrast, the expression of MMP-1 mRNA and protein in grafted veins decreased significantly on days 14 and 28, whereas the expression of TIMP-1 mRNA and protein increased significantly in grafted veins on days 14 and 28 compared to control veins (*Ps* <0.05 for all).

### Nanoparticle-mediated Delivery of a TGF-β1 Antisense-expressing Construct Down-regulates the Expression of TGF-β1 mRNA and Protein in Grafted Veins

The impact of nanoparticle-mediated delivery of a *TGF-β1* antisense-expressing construct on the expression of TGF-β1 mRNA and protein in grafted veins was evaluated by RT-PCR and Western blotting, respectively. As shown in Figure 5, treatment with a *TGF-β1* antisense-expressing construct significantly down-regulated the expression of TGF-β1 mRNA and protein in grafted veins. Immunohistochemistry was performed in order to examine the effect that delivery of the *TGF-β1* antisense-expressing construct has on MMP1 and TIMP1 protein expression. The results show that nanoparticle-mediated delivery of the *TGF-β1* antisense-expressing construct increases the expression of MMP1 protein but decreases the expression of TIMP1 protein (Table 3, Fig. 6).

### Nanoparticle-mediated Delivery of a TGF-β1 Antisense-expressing Construct Inhibits Intimal Hyperplasia in Grafted Veins

pMAMneo-Anti-*TGF-β1*-loaded nanoparticles were uniform and spherical, with a diameter of about 50–100 nm. The concentration of DNA loaded on the nanoparticles was 5.3%. Two weeks after delivery, the intima thickened significantly in grafted veins treated with normal saline or an empty vector (Fig. 7). In contrast, intimal thickening was significantly suppressed in grafted veins treated with the *TGF-β1* antisense-expressing construct. Furthermore, the intimal thickness was significantly decreased in grafted veins treated with the *TGF-β1* antisense-expressing construct than in those treated with normal saline or empty vector (20.5±4.7 µm vs. 47.3±6.4 µm, and 20.5±4.7 µm vs. 44.2±8.1 µm, respectively; *Ps* <0.001 for both).

## Discussion

In the present study, we examined the dynamic expression of TGF-β1, TGF-β RI, MMP-1, and TIMP-1 in grafted veins of a rat model. We have found that TGF-β1 was up-regulated prior to the development of intimal hyperplasia, and TGF-β R1 up-regulation was concomitant with the presence of intimal hyperplasia. We also discovered that MMP-1 was up-regulated and TIMP-1 down-regulated during intimal hyperplasia in grafted veins. In addition, we demonstrated that nanoparticle-mediated delivery of a *TGF-β1* antisense-expressing construct down-regulated TGF-β1 and TIMP1 expression, up-regulated MMP-1 expression, and inhibited intimal hyperplasia in grafted veins.

Numerous *in vitro* and *in vivo* studies have correlated TGF-β1 with intimal thickening [6]. After vascular injury, TGF-β1 is highly expressed in the intimal regions in both animal models and human specimens [18], [19]. Treatment with recombinant TGF-β1 or enhanced local expression of TGF-β1 results in significantly increased intimal thickening [9], [10], whereas direct or indirect inhibition of TGF-β1 activity attenuates intimal thickening and vascular restenosis [3], [11]–[13]. In our study, TGF-β1 was up-regulated prior to the appearance of intimal hyperplasia, and the down-regulation of TGF-β1 expression inhibited intimal hyperplasia in grafted veins. These results provide further evidence that TGF-β1 is a critical regulator of intimal hyperplasia after vascular injury.

The up-regulation of TGF-β1 after vascular injury results in the activation of various downstream pathways. Active TGF-β1 initiates cell signaling by binding to TGF-β1 receptor type II (TGF-β RII), which then recruits and dimerizes with TGF-β1 R1 forming a heterotrimeric complex. Formation of this heterotrimeric complex induces the serine-threonine kinase activity of these receptors and stimulates signaling via the Smad pathway [20]. In the present study, we found that TGF-β RI expression was up-regulated concomitantly with intimal hyperplasia, suggesting the involvement of this receptor in the development of intimal hyperplasia in grafted veins. Consistent with our finding, a previous study showed that inhibiting binding of active TGF-β1 to TGF-β RII prevented TGF-β1-mediated intimal hyperplasia [3].

ECM proteins constitute approximately 90% of the intimal mass in restenotic lesions [1]. TGF-β1 up-regulation leads to luminal narrowing in grafted veins, not only by promoting VSMC proliferation and migration, but also by stimulating ECM protein expression. Inhibition of TGF-β1 expression suppresses VSMC proliferation and migration and ECM synthesis [6]. It has been shown that TGF-β1 promotes the synthesis of collagen, fibronectin, and proteoglycans, and stimulates VSMC proliferation and migration during intimal hyperplasia [6], [21], [22]. MMPs and TIMPs can act together to regulate both degradation of the major components of the vascular ECM as well as VSMC proliferation and migration [4], [5]. The coordinated regulation of MMPs and TIMPs has been documented to contribute to intimal hyperplasia after vascular injury [4]. There is evidence that increased TGF-β1 expression leads to local increases in MMPs in vascular endothelial cells and VSMCs [23]. In our study, MMP-1 and TIMP-1 expression was temporally related to intimal expansion, and nanoparticle-mediated delivery of the TGF-β1 antisense-expressing construct increased MMP1 protein expression, but decreased the expression of TIMP1 protein. These data suggest that TGF-β1 controls intimal hyperplasia through the regulation of MMP1 and TIMP1 expression. Interestingly, increased MMP expression may also augment the bioavailability of TGF-β1, creating a feedback loop promoting intimal thickening [23].

Since TGF-β1 plays a critical role in promoting intimal hyperplasia, it represents an appropriate target for treatment and prevention of stenosis or occlusion of grafted veins. TGF-β1 synthesis, activation, and downstream regulators may serve as treatment targets. The use of antibodies [11], inhibitors [24], antisense oligonucleotides [25], soluble receptor [3], [26], and ribozymes [27] has proven effective in preventing luminal stenosis in animal models. They offer the promise of preventing vascular restenosis while simultaneously limiting systemic side effects. However, further human studies are required to determine the clinical utility of the different strategies for TGF-β1 inhibition.

Several clinical trials have demonstrated that oral administration of TGF-β1 inhibitors failed to suppress intimal thickening after arterial injury, indicating that the mode of treatment delivery may greatly affect the efficacy of therapeutics directed towards intimal thickening [6], [15]. Moreover, orally delivered medications are associated with systemic side effects [28]. Local drug delivery might be a more effective way to obtain higher tissue drug levels at the injury site and decrease the potential adverse systemic drug-associated side effects [16]. However, traditional local delivery systems have the disadvantage of very low tissue uptake and lack of sustained delivery with rapid washout of the agent. Nanoparticles have been investigated for the delivery of various types of therapeutic agents, including proteins, peptides, and DNA [29]. In addition to providing sustained release, nanoparticles can protect the encapsulated agent from enzymatic degradation. Moreover, unlike most of the cationic polymers and lipids that are commonly used for gene transfection, nanoparticles have no adverse affect on cell viability and are well tolerated by the body and cells. Furthermore, due to the small size of nanoparticles there is no risk of arterial occlusion [30]. For these reasons, nanoparticles may be used to target drugs or genes to the endothelium to achieve sustained therapeutic effects. Several studies have shown that various nanoparticle formulations can reduce TGF-β1-induced intimal thickening and restenosis in animal models [31], [32]. In our study, nanoparticle-mediated delivery of a *TGF-β1* antisense-expressing construct down-regulated the expression of TGF-β1 mRNA and protein and significantly inhibited intimal hyperplasia in grafted veins, demonstrating the feasibility of this strategy to target TGF-β1-induced intimal thickening.

In conclusion, we have demonstrated that expression of TGF-β1, TGF-β RI, MMP-1, and TIMP-1 is temporally related to intimal thickening in grafted veins. Taken together, these results suggest that TGF-β1 controls intimal hyperplasia via TGF-β RI possibly by regulating the expression of MMP-1 and TIMP-1. We have also shown that nanoparticle-mediated delivery of a *TGF-β1* antisense-expressing construct down-regulates the expression of TGF-β1 and inhibits intimal hyperplasia in grafted veins. Our findings provide further evidence that TGF-β1 plays an integral role in the development of intimal hyperplasia after vascular injury. Finally, we show that nanoparticle-mediated delivery of TGF-β1 antisense-expressing constructs is a feasible strategy to prevent TGF-β1-induced intimal thickening.
